# Assessment of Using the Syndromic Approach in Managing Patients With Sexually Transmitted Diseases Among the National Guard Primary Health Care Physicians, Jeddah City, Saudi Arabia

**DOI:** 10.7759/cureus.21502

**Published:** 2022-01-22

**Authors:** Hani S Almugti, Ruqaiyah N Al Hakeem, Ahmed M Alghamdi, Sarah A Aldamen, Abdullah A Alfaifi, Salam Algharbi, Mohammed A Al-Shehri, Essa M Atafi, Abdullah Al Rashidi, Norah Alturki, Abdullah S Al Amer, Shahd O AlMarei, Abdulaziz A AL Hunaiti, Asma S Al Shabragi, Asmaa S Al Barakati

**Affiliations:** 1 Ministry of National Guard Health Affairs, King Abdullah International Medical Research Center, King Saud Bin Abdulaziz University for Health Sciences, Jeddah, SAU; 2 Family Medicine, Faculty of Medicine, King Faisal University, Al-Ahsa, SAU; 3 Family Medicine, Faculty of Medicine, Al-Baha University, Al Bahah, SAU; 4 Pharmacology and Therapeutics, Faculty of Pharmacy, Mohammed AlMana College for Medical Sciences, Dammam, SAU; 5 Microbiology and Immunology, Faculty of Clinical Laboratory Sciences, King Abdulaziz University, Jeddah, SAU; 6 Pharmacology and Therapeutics, Faculty of Pharmacy, University of Hail, Hail, SAU; 7 Pharmacology and Therapeutics, Faculty of Clinical Pharmacy, Al-Baha University, Al Bahah, SAU; 8 Laboratory Medicine, College of Applied Medical Sciences, King Khalid University, Abha, SAU; 9 Family Medicine, Faculty of Medicine, Jordan University of Science and Technology, Amman, JOR; 10 Medicine, Faculty of Medicine, King Saud University, Riyadh, SAU; 11 Family Medicine, Faculty of Medicine, Jazan University, Jazan, SAU; 12 Family Medicine, Faculty of Medicine, King Abdulaziz University, Jeddah, SAU; 13 Pharmacology and Therapeutics, Faculty of Pharmacy, King Khalid University, Abha, SAU; 14 Family Medicine, Medical University of Silesia, Katowice, POL

**Keywords:** saudi arabia, syndromic approach, sexually transmitted diseases, physicians, primary health care

## Abstract

Background

Sexually transmitted infections (STIs) include a group of clinical syndromes that can be transmitted mainly through sexual activity. Using STIs’ syndromic approach for diagnosis and management is widely recommended to control and reduce the burden of these transmissible diseases.

Objective

The objective of this article is to assess the knowledge and practice of physicians concerning syndromic management of STIs in National Guard Primary Health Care (PHC) centers in Jeddah city, Saudi Arabia.

Materials and methods

This observational study was conducted at the National Guard PHC centers in Jeddah City, Saudi Arabia. An interview-administered questionnaire was designed. Fifty physicians have met the inclusion criteria, and all of them were included in the present study.

Results

Of the study population, 47 PHC physicians (response rate was 94%) were interviewed and the questionnaire was completed. Overall, the physicians' knowledge was different from one syndrome to another; it was highest for urethral discharge (72%) and lowest for vaginal discharge in pregnant women (21%). During the last 10 days, the physicians in the present study reported that two-thirds of their cases of STI were urethral discharge cases. However, during the previous 10 days, the practice assessment revealed that most physicians (76%) were correctly prescribed the medications as indicated by specific patients' syndromes.

Conclusion

Syndromic management is essential guidance to control and reduce the burden of STIs. Overall knowledge and practice of physicians were different from one syndrome to another. There is a need to design continuing medical education programs targeting PHC physicians to be clinically and culturally competent against socially sensitive diseases like STIs.

## Introduction

Sexually transmitted diseases (STDs), also known as sexually transmitted infections (STIs), include a group of clinical syndromes that can be transmitted mainly through sexual activity [1]. Several types of pathogens, including bacteria, viruses, and parasites, are the causative agents behind the following common STIs: chlamydia infection, gonorrhea, syphilis, human papillomavirus infection (genital warts), trichomoniasis, chancroid, genital herpes, hepatitis B infection, and human immunodeficiency virus (HIV) infection [2]. World Health Organization (WHO) considers STIs as a significant public health challenge with a bad impact on the quality of life and may lead to serious complications and death [2].

Globally, more than one million curable STIs are recognized every day as one of the top five reasons to visit health care services in developing countries [3]. Data from WHO collected from 2009 to 2016 showed that the incident cases of STIs were 376.4 million in the age group between 15 and 49 years, slightly half of all these cases were from gonorrhea and chlamydia [3]. In Saudi Arabia, 68,886 new cases of STIs were surveyed from 2005 to 2012 in a cross-sectional study; among all, the most prevalent causes of STIs were urethritis, trichomoniasis, and HIV [4]. Another Saudi study revealed that vaginal discharge and lower abdominal pain were the most frequent symptoms of STIs that urge patients to seek health care [5].

Primary health care (PHC) centers face the majority of symptoms of STIs. The combined health services of prevention and treatment are the global strategy for preventing and controlling STIs. According to the Saudi Central Board for Accreditation of Healthcare Institutes (CBAHI), one of the essential standards to increase the quality of health service in PHC is the preventive efforts against STIs that should include well-designed programs of health education, vaccine administration, and screening [6]. Research indicated that the conventional approaches to STI diagnosis and treatment struggle with the challenges of limited recourses, delayed diagnosis [7], and difficult access to treatment [8,9]. Delay in treating the cases is associated with severe complications such as pelvic inflammatory disease, infertility, ectopic pregnancy, abortions, fetal loss and congenital infections, and cancer [6]. Moreover, the risk of acquisition of HIV from HIV cases will increase if the STI cases are not well treated, thus resulting in a high prevalence of HIV cases and associated complications [2].

The syndromic approach for diagnosing and managing STIs is recommended by WHO to minimize the delay in the treatment. PHC physicians are the cornerstone to implementing such an approach. However, different studies conducted in developing countries showed poor knowledge [10,11] and practice [12] among the health care workers regarding the syndromic management of STIs. Still, no data is known from Saudi Arabia. So the present study highlighted the effectiveness and clinical advantages of using the STIs’ syndromic approach for diagnosis and management among the National Guard PHC centers physicians.

## Materials and methods

Objective

The objective is to assess the knowledge and practice of the National Guard PHC physicians concerning syndromic management of STIs in Jeddah city, Saudi Arabia.

Study design

The research design was a cross-sectional study.

Study setting

The study was conducted at the National Guard PHC centers of Jeddah city, Saudi Arabia. There are three main PHC centers located in Jeddah city: Specialized Polyclinic Center, Iskan Jeddah Center, Bahrah Center.

Population

The study recruited staff of physicians working at the National Guard PHC centers of Jeddah city. However, we have applied some inclusion and exclusion criteria as follows.

Inclusion criteria

Physicians, regardless of their gender or nationality, who are working in National Guard PHC centers of Jeddah city and physicians who are covering the general or family medicine clinics were included in this study.

Exclusion criteria

Trainee physicians (medical students, medical interns, and residents) were excluded. 

Sample size and sampling

Through communication with the office of the Deputy Executive Director of PHC western region, a list was obtained that included the physicians who currently work at the National Guard PHC centers of Jeddah city. After applying the inclusion and exclusion criteria, 50 physicians who met the inclusion criteria were included in the present study.

Operation definition

Variables

Dependent variables: Knowledge and practice of physicians were the dependent variables.

Independent variables: Age of physicians, gender of physicians, length of practice, medical specialty, obtaining training courses in caring and dealing with STI patients were the independent variables.

Knowledge and Practice of Physicians

The knowledge of physicians was measured by how correctly the physicians, according to the National Guideline, answered the questions of drug prescription details (name of drug-dose-frequency-duration) for each syndrome of STIs (urethral discharge, vaginal discharge, and genital ulcer).

The practice of physicians was measured according to the National Guideline by how correctly the physicians reported the drug prescription details in the patients' file during the past 10 days (name of drug-dose-frequency-duration) for each of the STI syndromes (urethral discharge, vaginal discharge, and genital ulcer). The additional assessment for the current clinical practice was conducted by using closed-end questions regarding the physicians' experience in treating STI cases.

Data collection tool

Data was collected using an interview-administered questionnaire adopted from the District STI Quality of Care Assessment (DISCA) tool [13]. The questionnaire is revised by an expert panel of public health consultants, family and preventive medicine physicians, health informatics, and health administration to ensure face and content validity. The Cronbach's alpha coefficient was 0.8, indicating good reliability. Each section of the questionnaire is provided in Appendix A.

Data collection was continued for a duration of two months (September and October of 2021) after obtaining approval (NRJ21J/254/10) from the Ethical and Scientific Committee of King Abdullah International Medical Research Center at King Abdulaziz Medical City. Through the interview-administered questionnaire, the data were collected. Fortunately, National Guard health affairs currently have BEST Care's digital health care system [14] that helped us collect data.

Four of the current study researches were assigned to assess each physician's knowledge and practice (there were scheduled visits of date and time for each physician). After answering the knowledge questions by the physician, every physician shared with the researchers his/her health care plan from his/her account in the best care system that belonged to one patient seen by a physician during the last 10 days. Every researcher had his/her checklist for every physician, and after finishing their checklists of each visit, they were required to submit the final assessment form, which was approved by all of them. We scrupulously guarded the confidentiality of physicians and their patients' data throughout the study. Consent to participate in the present study and for publication was obtained from the participants.

Data management and statistical analysis

The Statistical Package for the Social Sciences (SPSS) software for Windows was used (version 20.0; IBM Corp., Armonk, NY, USA). Quality control was performed at the coding and data entry stages. Data were presented using descriptive statistics in frequencies and percentages for qualitative variables and means and standard deviations for quantitative variables. Chi-square test and Fisher's exact test were used to record the statistical significance between participants' answers and their medical specialty, obtaining specific training for management of STIs and length of the clinical practice.

## Results

Characteristics of the study subjects

Of the study population (the response rate was 94%), 47 PHC physicians were interviewed and completed the questionnaire. Their mean age was 39 years ranging between 25 and 58 years. As shown in Table 1, almost half of the physicians were board-certified and had more than 10 years of medical experience and practice.

**Table 1:** General characteristics of the participants (n = 47)

Demographic Characteristics	Frequency (n)	Percent (%)
Age (years)		
25-30	4	8.5
31-40	21	44.7
41-50	13	27.7
More than 50	9	19.1
Range	25-58 years	
Mean ± SD	39 ± 8 years	
Gender		
Male	25	53
Female	22	47
Level of education		
Bachelor	16	34
Master degree	4	8
Board-certified or Ph.D. degree	22	47
Fellowship	5	11
Years of experience		
0-5	9	19
6-10	13	28
More than 10	25	53

**Figure 1:**
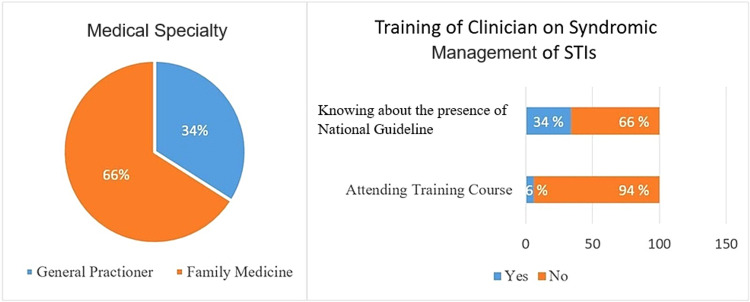
Percentage of primary health care physicians among different medical specialties and percentage of trained physicians on syndromic management of STIs STIs, Sexually transmitted infections.

Figure 1 shows that two-thirds of physicians are certified as family medicine physicians, and only six percent of them had training courses in caring for and dealing with STI patients. Moreover, one-third of participants reported that they were aware of and received the National Guidelines of Syndromic Management of STIs issued by the Ministry of Health.

Assessment of physicians' knowledge

According to the national syndromic approach for the management of STIs, data in Table 2 indicate that physicians who correctly reported the name of the drugs for urethral discharge management were 42 (89.4%) and physicians who mentioned the dosage, frequency, and duration correctly were 34 (72%). For the vaginal discharge management, the physicians who correctly mentioned the drugs' name were 45 (96%), and the physicians who mentioned the dosage, frequency, and duration correctly were 24 (51%). Out of all physicians, those who correctly reported the name of the drugs for genital ulcer management were 42 (89.4%), and only one-third of them (30%) mentioned the related dosage, frequency, and duration correctly. Furthermore, for the management of pregnant women with vaginal discharge, physicians who correctly named the drugs were 30 (64%), and physicians who mentioned related dosage, frequency, and duration correctly were 10 (21%).

**Table 2:** Physicians' knowledge of STI syndromes' treatment *Statistically significant at p < 0.05. GP, General Practice; FM, Family Medicine; STIs, sexually transmitted infections.

Knowledge of Syndromes		Medical Specialty		Had Training on Syndromic Management of STIs	
		GP (n)	FM (n)	No (n)	Yes (n)
Urethral discharge	Correct drug				
	No (n)	0	5	5	0
	Yes (n)	16	26	39	3
	P-value	0.08		0.5	
	Correct prescription				
	No (n)	2	11	13	0
	Yes (n)	14	20	31	3
	P-value	0.09		0.26	
Vaginal discharge	Correct drug				
	No (n)	0	2	2	0
	Yes (n)	16	29	42	3
	P-value	0.299		0.7	
	Correct prescription				
	No (n)	9	14	23	0
	Yes (n)	7	17	21	3
	P-value	0.47		0.08	
Genital ulcer	Correct drug				
	No (n)	0	5	5	0
	Yes (n)	16	26	39	3
	P-value	0.08		0.53	
	Correct prescription				
	No (n)	7	26	33	0
	Yes (n)	9	5	11	3
	P-value	0.004*		0.006*	
Pregnant women with vaginal discharge	Correct drug				
	No (n)	8	9	14	0
	Yes (n)	8	22	30	3
	P-value	0.15		0.017*	
	Correct prescription				
	No (n)	14	23	34	0
	Yes (n)	2	8	10	3
	P-value	0.29		0.35	

Together, these results provide important insights into different levels of physicians' knowledge against the prescription details of syndrome type; it was highest for urethral discharge (72%) and lowest for vaginal discharge in pregnant women (21%). The association between physicians' knowledge and medical specialty is interesting because the physicians' knowledge of prescribing treatment for genital ulcers among the general practitioner is higher than the family medicine physicians with statistical significance (p < 0.05). However, there is no statistical significance among other syndromes of STIs with medical specialties.

A clear benefit of obtaining specific training for the management of STIs could not be identified in the present analysis due to the small number of physicians (three of 47) who had this training. However, the physicians who had received training in the management of STIs had higher knowledge of prescribing the treatment compared with the physicians who did not, especially among the syndrome of genital ulcer and pregnant with vaginal discharge (p < 0.05).

Further statistical tests in Table 3 revealed that compared with physicians who had fewer years of experience, the physicians with more than 10 years of experience were correctly reporting the name of the drugs, dosage, frequency, and duration of treatment. However, there was only one statistical significance between the physicians with more than 10 years of experience and the good knowledge of genital ulcer syndrome treatment details regarding the dosage, frequency, and duration. There were no significant differences between the physicians' knowledge of other STI syndromes' treatment and their length of experience.

**Table 3:** Relation between physicians' knowledge of STI syndromes' treatment and their length of experience *Statistically significant at p < 0.05. STI, Sexually transmitted infection.

Knowledge of Syndromes		Length of Experience		
		0–5 (Years)	6–10 (Years)	>10 (Years)
Urethral discharge	Correct drug			
	No (n)	0	0	5
	Yes (n)	9	13	20
	P-value	0.08		
	Correct prescription			
	No (n)	2	2	9
	Yes (n)	7	11	16
	P-value	0.37		
Vaginal discharge	Correct drug			
	No (n)	0	0	2
	Yes (n)	9	13	23
	P-value	0.4		
	Correct prescription			
	No (n)	7	5	11
	Yes (n)	2	8	14
	P-value	0.149		
Genital ulcer	Correct drug			
	No (n)	0	2	3
	Yes (n)	9	11	22
	P-value	0.49		
	Correct prescription			
	No (n)	7	13	13
	Yes (n)	2	0	12
	P-value	0.008*		
Pregnant women with vaginal discharge	Correct drug			
	No (n)	3	4	10
	Yes (n)	6	9	15
	P-value	0.83		
	Correct prescription			
	No (n)	7	13	17
	Yes (n)	2	0	8
	P-value	0.073		

Assessment of physicians' practice

**Figure 2:**
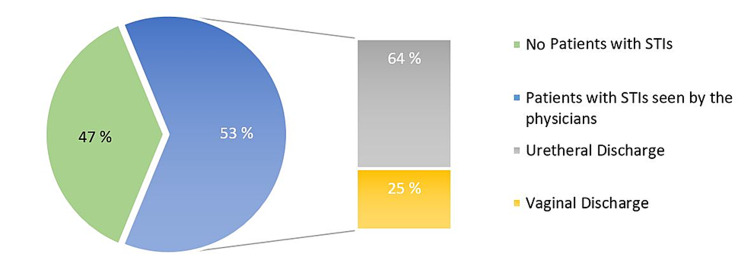
Percentage of physicians who treated patients with STIs in the last 10 days (n = 47) STIs, Sexually transmitted infections.

As shown in Figure 2, 53% of physicians treated cases of STIs in the last 10 days. Upon reviewing the electronic medical files of those patients, urethral discharge cases were the highest incidence cases for about two-thirds of all cases, vaginal discharge came in the second of the list by 25%, and genital ulcer in 11% of cases. Moreover, the majority of physicians (76%) were correctly prescribed the medication of related STIs' syndrome for their patients.

**Figure 3:**
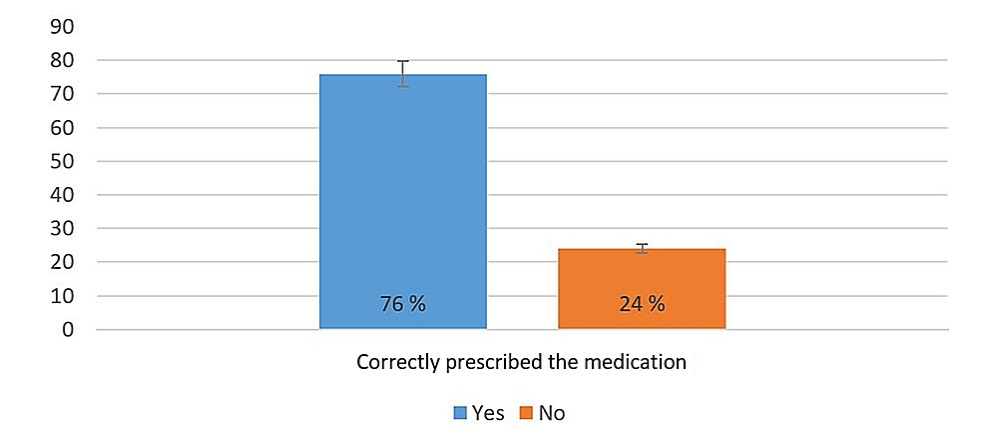
Percentage of physicians from the medical record review who correctly prescribed the medication for their STI patients in the last 10 days (n = 25) STIs, Sexually transmitted infections.

**Figure 4:**
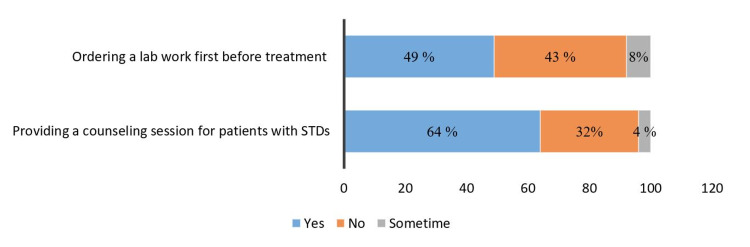
Percentage of physicians' responses against clinical practice questions (n = 47) STDs, Sexually transmitted diseases.

Concerning the general practice against STIs cases, from Figure 3*,* we can see that two-thirds of physicians used to provide health counseling for their patients diagnosed with STIs. On the other hand, Figure 4 shows that half of the physicians would usually confirm the diagnosis by lab investigations before starting the treatment.

**Figure 5:**
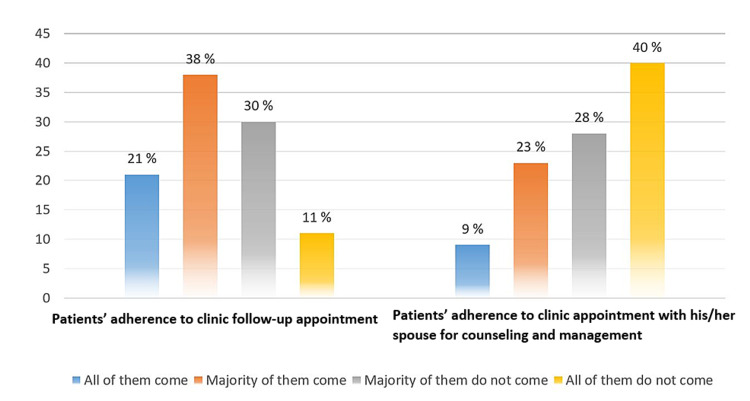
Percentage of physicians' responses against questions of their patients' adherence to management plan (n = 47)

Moreover, in Figure 5, physicians experienced that 41% of their patients had low adherence to follow-up appointments and two-thirds of them reported non-show in follow-up appointments with the patients' spouses for treatment and counseling purposes.

## Discussion

The syndromic approach is widely used as a problem-oriented process (based on the patient's symptoms) in managing patients with STDs [6]. The present study assessed the knowledge and practice of physicians concerning syndromic management of STIs in National Guard PHC centers in Jeddah city, Saudi Arabia. Although caring for STIs is a teamwork effort from different health specialties of nurses and other admin staff, the present study targeted the physicians as they are the case managers in providing diagnosis, treatment, and counseling.

Overall, the findings of this study suggested a higher level of knowledge toward the correct prescription details for the syndromes of urethral discharge and vaginal discharge; however, a lower level of knowledge was observed in syndromes of vaginal discharge in pregnant women and genital ulcers. These differences in knowledge among syndromes might be attributed to the variation in their prevalence rate, thus giving more experience in correctly managing these symptoms. In addition, half of the participants were exposed to the highly prevalent syndrome for more than 10 years, and most physicians (two-thirds) are certified with post-graduation certificates. However, the present data is higher than the results of two previous studies in Pakistan and Namibia that were conducted to assess general practitioners' knowledge, where the knowledge of the recommended treatment regimen was 55% and 56%, respectively [11,15].

Although only 30% of physicians were aware of the guidelines for the syndromic management of STIs, clinically, the current study showed that the majority of physicians (76%) correctly prescribed the medication concerning the STIs' syndrome for their patients in the last 10 days. These findings go in line with the results of previous studies [10,11] that explained this finding by most physicians following their clinical flowchart in treating STI cases. However, our result is higher than the findings in these studies [11,16]; this might be because most participants had more than 10 years of experience with a high level of education.

The syndromic management of STIs consists of the comprehensive approach that includes treating the case, providing clinical counseling, and preventing the further spread of STIs by treating the partners. Behavioral counseling helps to increase the level of knowledge and guide patients for safer sex practices [17]. Clinically, the most crucial relevant finding was that almost one-third of physicians were not providing health counseling for their patients diagnosed with STIs. However, the United States Preventive Services Task Force (USPSTF) recommended high-intensity behavioral counseling interventions in patients at increased risk for STIs as it effectively reduces future STI acquisition by 30% [17,18].

Considering the cultural and social aspects, STIs are sensitive topics, and diagnosed patients are frequently stigmatized in Saudi culture [19]. All of that preclude patients from seeking help, delaying treatment, and prohibiting the effective treatment of the partners. The current study's findings expected and showed that physicians reported 41% of their patients having low adherence to follow-up appointments and two-thirds of them missing the follow-up appointment with the patients' spouses for treatment and counseling purposes. These findings may help us understand that in addition to the treatment regimen, primary healthcare physicians have to be taught about the importance of being culturally competent against sensitive diseases like STI and the cost-effectiveness of providing clinical counseling.

Finally, the current study has only examined PHC centers in Jeddah city, and the results cannot enhance the generalizability. To support the power of the study, national-based studies could be helpful to collect a larger sample. Despite these limitations, our study addresses the knowledge and practice of physicians concerning syndromic management of STIs in National Guard PHC centers in Jeddah city, Saudi Arabia.

## Conclusions

STIs' syndromic management was designed to control and reduce the burden of these transmissible diseases. This is a rare study in Saudi Arabia aimed to assess the use of this approach among PHC physicians. Overall knowledge and practice of physicians were different from one syndrome to another. The findings of this study have several important implications for future practice. Efforts should turn to include the management of STIs as part of continuing medical education programs. Considerably, more work will need to be done from the leaderships to enhance the effectiveness of clinical practice by widely disseminating the clinical pathway and flow chart of the management steps.

## Appendices

The questionnaire of this study consisted of three sections, which are as follows.

First section

It included seven socio-demographic questions (age, gender, medical specialty, length of practice, and had training courses for syndromic management of STIs or not).

Second section

It was the knowledge assessment that consisted of closed-ended questions, and all physicians' answers were assessed according to the guidelines of syndromic management of STIs as follows:

A patient comes with urethral discharge; what are the correct drug, correct dose, correct frequency, and correct duration?

A patient comes with vaginal discharge; what are the correct drug, correct dose, correct frequency, and correct duration?

A patient comes with a genital ulcer; what are the correct drug, correct dose, correct frequency, and correct duration?

For pregnant women with vaginal discharge, what are the correct drug, correct dose, correct frequency, and correct duration?

Do you have an idea if there is a national guideline for the syndromic management of STIs issued by the Ministry of Health?

Third section

It was the practice assessment that consisted of closed-ended questions, and the answers were assessed according to the guidelines of syndromic management of STIs after reviewing the physicians' report in each patient's file as follows:

In the last 10 days, did you see any patient with one of the following symptoms; urethral discharge or vaginal discharge or genital ulcer?

What was the symptom?

What was the drug?

What was the dose?

What was the frequency of the drug?

What was the duration?

Did you document the counseling session?

Generally, do you provide a counseling session for patients with STDs?

Generally, do you request lab work first before starting the treatment?

From your experience, patient with STI usually adheres to clinic appointment and attends follow-up appointments?

From your experience, patient with STI usually adheres to clinic appointments with her/his spouse for counseling and management?

## References

[1] Workowski KA, Bolan GA (2015). Sexually transmitted diseases treatment guidelines, 2015. MMWR Recomm Rep.

[2] (2021). Sexually transmitted infections (STIs). https://www.who.int/news-room/fact-sheets/detail/sexually-transmitted-infections-(stis).

[3] Rowley J, Vander Hoorn S, Korenromp E (2019). Chlamydia, gonorrhoea, trichomoniasis and syphilis: global prevalence and incidence estimates, 2016. Bull World Health Organ.

[4] Memish ZA, Filemban SM, Al-Hakeem RF, Hassan MH, Al-Tawfiq JA (2016). Sexually transmitted infections case notification rates in the Kingdom of Saudi Arabia, 2005-2012. J Infect Dev Ctries.

[5] Kabbash IA, Al-Mazroa MA, Memish ZA (2011). Evaluation of syndromic management of sexually transmitted infections in Saudi Arabia. J Infect Public Health.

[6] World health organization (2021). Global health sector strategy on sexually transmitted infections, 2016-2021. https://www.who.int/reproductivehealth/publications/rtis/ghss-stis/en/.

[7] Shrivastava SR, Shrivastava PS, Ramasamy J (2014). Utility of syndromic approach in management of sexually transmitted infections: public health perspective. J Coast Life Med.

[8] Liu H, Jamison D, Li X, Ma E, Yin Y, Detels R (2003). Is syndromic management better than the current approach for treatment of STDs in China? Evaluation of the cost-effectiveness of syndromic management for male STD patients. Sex Transm Dis.

[9] Tsai CH, Lee TC, Chang HL, Tang LH, Chiang CC, Chen KT (2008). The cost-effectiveness of syndromic management for male sexually transmitted disease patients with urethral discharge symptoms and genital ulcer disease in Taiwan. Sex Transm Infect.

[10] Alemayehu A, Godana W (2015). Knowledge and practice of clinicians regarding syndromic management of sexually transmitted infections in public health facilities of gamo gofa zone, South Ethiopia. J Sex Transm Dis.

[11] Hussain MF, Khanani MR, Siddiqui SE, Manzar N, Raza S, Qamar S (2011). Knowledge, attitudes & practices (KAP) of general practitioners (GPS) regarding sexually transmitted diseases (STDS) and HIV/AIDS in Karachi, Pakistan. J Pak Med Assoc.

[12] Adhikari C, Sherchan L, Thapa SB, Adhikari LM (2014). Effectiveness of syndromic STI case management/RH training in knowledge and practice of auxiliary health workers. Journal of Universal College of Medical Sciences.

[13] Bachmann MO, Colvin MS, Nsibande D, Connolly C, Curtis B (2004). Quality of primary care for sexually transmitted diseases in Durban, South Africa: influences of patient, nurse, organizational and socioeconomic characteristics. Int J STD AIDS.

[14] (2021). Ministry of National Guard, National Guard Health Affairs, Hospital Information System “BESTCare”. https://ngha.med.sa/English/AboutNGHA/bestcare/Pages/Default.aspx.

[15] Iipinge SN, Pretorius L (2012). The delivery and quality of sexually transmitted infections treatment by private general practitioners in Windhoek Namibia. Glob J Health Sci.

[16] Bitera R, Alary M, Mâsse B (2002). Quality of disease management of sexually transmitted diseases: investigation of care in six countries in West Africa. Sante.

[17] LeFevre ML (2014). Behavioral counseling interventions to prevent sexually transmitted infections: U.S. PreventiveServices Task Force recommendation statement. Ann Intern Med.

[18] O'Connor EA, Lin JS, Burda BU, Henderson JT, Walsh ES, Whitlock EP (2014). Behavioral sexual risk-reduction counseling in primary care to prevent sexually transmitted infections: a systematic review for the U.S. Preventive Services Task Force. Ann Intern Med.

[19] Badahdah AM (2010). Stigmatization of persons with HIV/AIDS in Saudi Arabia. J Transcult Nurs.

